# Loss of MYC and E-box3 binding contributes to defective MYC-mediated transcriptional suppression of human MC-let-7a-1~let-7d in glioblastoma

**DOI:** 10.18632/oncotarget.10517

**Published:** 2016-07-09

**Authors:** Zifeng Wang, Sheng Lin, Ji Zhang, Zhenhua Xu, Yu Xiang, Hong Yao, Lei Ge, Dan Xie, Hsiang-fu Kung, Gang Lu, Wai Sang Poon, Quentin Liu, Marie Chia-mi Lin

**Affiliations:** ^1^ Sun Yat-sen University Cancer Center, State Key Laboratory of Oncology in South China, Collaborative Innovation Center of Cancer Medicine, Guangzhou, China; Institute of Cancer Stem Cell, Dalian Medical University, Dalian, China; ^2^ Shenzhen Key Lab of Translational Medicine of Tumor, School of Medicine, Shenzhen University, Shenzhen, China; ^3^ Brain Tumor Centre and Division of Neurosurgery, Department of Surgery, Faculty of Medicine, The Chinese University of Hong Kong, Prince of Wales Hospital, Shatin, Hong Kong, China; ^4^ Laboratory of Medical Genetics, Shenzhen Research Institute of Population and Family Planning, Shenzhen, China; ^5^ Biochemistry and Molecular Biology, Medical University of South Carolina, Charleston, SC, USA; ^6^ Jiangsu Eng. Lab of Cancer Biotherapy, Xuzhou Medical College, Xuzhou, China; ^7^ School of Biomedical Science, and State Key Laboratory in Oncology in South China, Chinese University of Hong Kong, Shatin, Hong Kong; ^8^ Department of Gastrointestinal Surgery, Tumor Hospital, Xinjiang Medical University, Urumqi, Xinjiang Uyghur Autonomous Region, China

**Keywords:** MC-let-7a-1~let-7d, MYC, glioblastoma, transcriptional regulation

## Abstract

Previously, we reported that MYC oncoprotein down-regulates the transcription of human MC-let-7a-1~let-7d microRNA cluster in hepatocarcinoma (HCC). Surprisingly, *in silico* analysis indicated that let-7 miRNA expression levels are not reduced in glioblastoma (GBM). Here we investigated the molecular basis of this differential expression. Using human GBM U87 and U251 cells, we first demonstrated that forced over-expression of MYC indeed could not down-regulate the expression of human MC-let-7a-1~let-7d microRNA cluster in GBM. Furthermore, analysis of MC-let-7a-1~let-7d promoter in GBM indicated that MYC failed to inhibit the promoter activity. Pearson's correlation and Linear Regression analysis using the expression data from GSE55092 (HCC) and GSE4290 (GBM) demonstrated a converse relationship of MC-let-7a-1~let-7d and MYC only in HCC but not in GBM. To understand the underlying mechanisms, we examined whether MYC could bind to the non-canonical E-box 3 located in the promoter of MC-let-7a-1~let-7d. Results from both chromatin immune-precipitation (ChIP) and super-shift assays clearly demonstrated the loss of MYC and E-box 3 binding in GBM, suggesting for the first time that a defective MYC and E-box3 binding in GBM is responsible for the differential MYC mediated transcriptional inhibition of MC-let-7a-1~let-7d and potentially other tumor suppressors. MYC and let-7 are key oncoprotein and tumor suppressor, respectively. Understanding the molecular mechanisms of their regulations will provide new insight and have important implications in the therapeutics of GBM as well as other cancers.

## INTRODUCTION

MicroRNAs (miRNAs) are short non-coding RNAs (~22 nt) that regulate protein expression and control diverse aspects of biology, including carcinogenesis [1]. The let-7 family miRNAs are down-regulated in various types of cancer including lung cancer, liver cancer, colon cancer, ovarian cancer, stomach cancer, leiomyoma and melanoma [2–15]. They have become a prototype miRNAs that function as tumor suppressors, as they inhibit the expressions of multiple oncogenes including Ras and MYC [16–19]. The human microRNA cluster *MC-let-7a-1~let-7d* consists of three members *let-7a-1, let-7f-1 and let-7d*. These three members together accounted for approximately 24% of the total let-7 precursors [15]. Hence, this cluster is a particularly important component of the let-7 family.

The MYC oncoprotein is a transcription factor which plays a pivotal role in carcinogenesis by transcriptional down-regulation of multiple tumor suppressors. MYC expression is significantly elevated in almost all cancers including HCC and GBM. We have previously demonstrated that MYC inhibits *MC-let-7a-1~let-7d* expression at transcriptional level through a non-canonical E-box 3 in HCC [15].

Both HCC and Glioblastoma (GBM, WHO grade IV, the most severe form of glioma) are highly invasive and destructive tumors with no effective treatment [20, 21]. Accumulated evidence has clearly shown the miRNAs play important roles in the carcinogenesis of HCC and GBM [15, 18, 22–26]. Using bioinformatics analysis of human miRNA expression data sets, we showed that the tumor suppressor *MC-let-7a-1~let-7d* is down-regulated in most types of cancer include HCC. Surprisingly, *in silico* analysis indicated that let-7 miRNA expression levels are not reduced in GBM [5, 27–35]. As MYC oncoprotein expression is elevated in both HCC and GBM, we investigated why MC-let-7a-1~let-7d expression is down-regulated in HCC but not in GBM.

Contrary to HCC HepG2 and L02 cells, we demonstrated that in human GBM U87 and U251 cells, forced over-expression of MYC could not down-regulate the expression of human MC-let-7a-1~let-7d microRNA cluster in GBM. In addition, we characterized MC-let-7a-1~let-7d promoter in GBM, and showed that MYC failed to inhibit the promoter activity of MC-let-7a-1~let-7d. As MYC down-regulates transcription mainly through the binding with a non-canonical E-box 3, we investigate the binding of MYC and the E-box 3 located in the promoter of MC-let-7a-1~let-7d in GBM. By both chromatin immune-precipitation (ChIP) and super-shift assays, we demonstrated the loss of MYC and E-box 3 binding. Taken together, these results revealed for the first time that GBM exhibited differential MYC mediated transcriptional inhibition on MC-let-7a-1~let-7d due to the defective MYC/E-box3 binding. MYC oncoprotein and MC-let-7a-1~let- 7d both play key roles in carcinogenesis. Understanding the molecular basis of their functions and regulations will provide new insight and important implications in the development of new therapeutics of GBM and other cancers.

## RESULTS

### The expression levels of let-7 miRNAs are not reduced in GBM

We first analyzed the expression level of 21 mature miRNA of let-7 family members in GBM as compared to normal brain tissues using the microarray data from 11 independent groups [27–35]. Results from ten groups indicated that let-7 miRNA levels are not significantly changed in GBM, and one group showed a modest down-regulation of only 3 miRNAs, let-7d, let-7f and let-7g (Supplementary Table S1).

**Figure 1:**
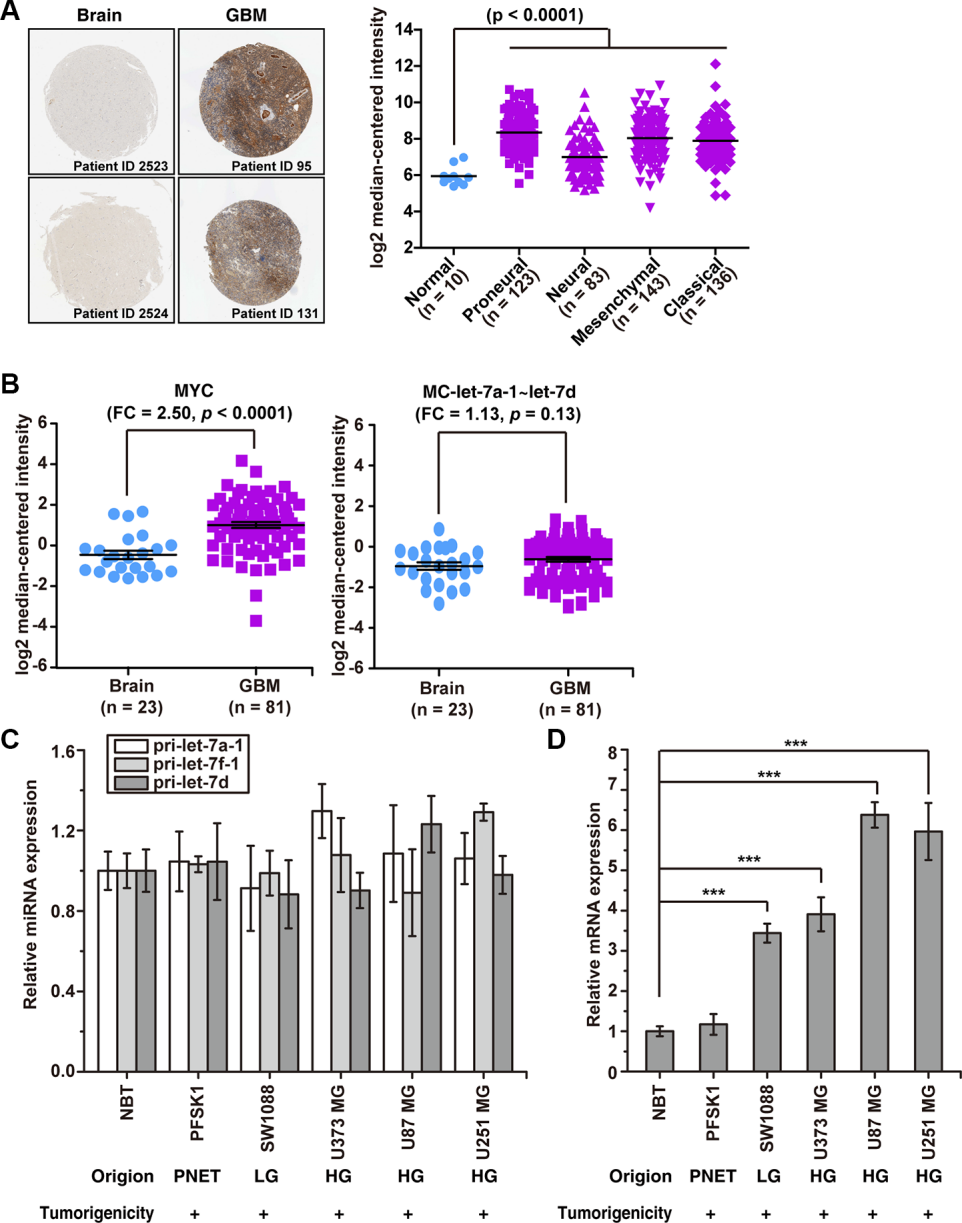
MC-let-7a-1~let-7d is not down-regulated, whereas Myc is up-regulated in GBM (**A**) left panel, the expression of c-Myc protein in normal brain tissues and GBM as determined by immunohistochemical staining obtained from Human Protein Atlas database. Right panel, the expression of c-Myc mRNA in normal brain tissues and GBM as determined by high throughput sequencing method from The Cancer Genome Atlas (TCGA) glioblastoma database. Patient specimens were categorized according to TCGA subtypes: classical, mesenchymal, proneural, and neural. (**B**) the expression levels of MC-let-7a-1~let-7d and MYC in normal brain tissues and GBM according to GEO Profile (GSE4290). FC, fold change.(**C**) the relative expression levels of primary MC-let-7a-1~let-7d members in normal human brain tissue (NBT), in human glioma cell lines (HG = highgrade; LG = low grade), and in a primitiveneuroectodermal tumor (PNET) cell line. (**D**) the relative expression levels of c-MycinNBT, in human glioma cell lines, and in PNETcell line. *p < 0.05; **p < 0.01; ***p < 0.001.

Next, we compared the expression level of precursor miRNA of let-7 family members in HCC/normal liver tissues and GBM/normal brain tissues. As shown in Table 1 (data from Pablo Landgraf. et al [5]), in 147 human cells and tissues, both the MC-let-7a-1~let-7d and the let-7 family precursors are significantly down-regulated (more than 10- fold reduction) in HCC. Surprisingly, these precursors are slightly up-regulated (less than 2-fold elevation) in GBM. The miR-21, miR-23a, miR-210 and miR-139, miR- 128 and miR-181 are listed as controls to shown that the deep sequence data (NGS) are consistent with the published results [27, 35–39]. The unbiased data-mining results suggest that MC-let-7a-1~let-7d is not down-regulated in GBM, in sharp contrast to its expression pattern in HCC. Consistent with the published reports, high level of MYC onco-protein expression in GBM was observed by immune-histochemical staining assays using The Human Protein Atlas database [40] (Figure 1A, left panel). Moreover, we also analyzed the expression of MYC in a large number of clinical glioblastoma specimens (*n* = 495) in The Cancer Genome Atlas (TCGA) glioblastoma database. Results indicated that MYC expression is significantly elevated in all glioblastoma subtypes relative to normal human cerebrum (Figure 1A, right panel).

**Table 1:** The ratio of let-7 in total miRNAs in GBM vs normal brain tissues (NBT) and HCC vs normal liver tissues (NLT)

miRNA/intracellular total miRNAs		NLT	HCC	Log FC	NBT	GBM	Log FC
MC-let-7a-1~let-7d		0.95%	0.33%	−1.55	1.45%	2.45%	0.75
let-7 family		4.23%	1.21%	−1.80	8.67%	16.57%	0.93
upregulated	miR-21	0.07%	22.35%	8.29	0.20%	10.96%	5.77
	miR-23a	0.11%	0.36%	1.75	0.10%	1.58%	3.98
	miR-210	0.00%	0.08%	> 1	0.02%	0.24%	3.60
downregulated	miR-139	0.07%	0.00%	< −1	0.34%	0.00%	< −1
	miR-128	0.00%	0.19%	> 1	2.94%	0.12%	−4.59
	miR-181	0.50%	0.19%	−1.39	6.13%	0.61%	−3.33

Annotation: The ratios are based on 147 human cells and tissues detected by Pablo Landgraf. et al. 2007. miR-21, miR-23a, miR-210 and miR-139, miR-128, miR-181 are listed to show that the deep sequence data (NGS) are consistent with the pubilished results. Log FC, log10-transformed fold change (tumor/normal).

To further confirm these findings, we carried out gene-expression analyses of MC-let-7a-1~let-7d and MYC in an independent gene-expression cohort including 81 GBM tissues and 23 normal brain tissues according to GEO Profile (GSE4290). The GPL570 microarray platform provides probes to detect the expression of let-7d host gene (MC-let-7a-1~let-7d). As shown in Figure 1B, while MYC level was significantly up-regulated 2.5-fold (*p* < 0.0001), the expression of MC-let-7a-1~let-7d was not significantly changed (1.13 fold, *p* = 0.13).

We further examined the expression of pri-let-7a-1, pri-let-7f-1, pri-let-7d and MYC in three human GBM cell lines from high-grade tumors (U-373 MG, U-87 MG, and U-251 MG), one low-grade glioma (SW-1088), and a malignant neuroectodermal tumor (PFSK-1) by Real-Time PCR assays. Consistently, the pri-let-7a-1, pri-let-7f-1, and pri-let-7d levels were not significantly changed as compared to normal brain tissue (NBT, Figure 1C), while MYC level was significantly elevated in all cell lines (Figure 1D).

### Characterization of the promoter of MC-let-7a-1~let-7d cluster in GBM U87 and U251 cells

**Figure 2:**
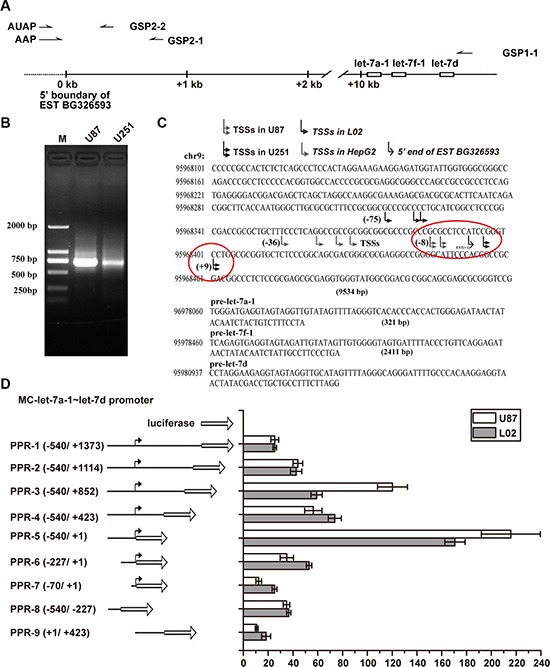
MC-let-7a-1~let-7d promoter characterization in GBM as compared to that in HCC (**A**) primers used for 5′RACE. Gene-specific primers (GSPs) GSP1-1 was used to synthesize the first strand cDNA. Primary amplification was carried out with abridged anchor primer (AAP) and GSP2-1 primer. Nested PCR was performed with abridged universal amplification primer (AUAP) and GSP2-2.(**B**) 5′RACE results. The ~700-bp products were amplified in U87 cells and U251 cells by using GSP2-2 and AUAP. (**C**) sequence analysis of 20 products from each cell lines revealed that variable TSSs were used. The major TSSs of MC-let-7a-1~let-7d in U87 is located about 28 bp, while in U251 is located about 40 bp downstream of the TSSs used in HepG2 cells. (**D**) schematic diagram of the reporter construct (left panel). Truncation of the downstream region to +1 dramatically improved the promoter activity, whereas truncation of the upstream region to −227 resulted in progressive loss of activity in both U87 cells and L02 cells.

Previously, we have characterized the MC-let-7a-1~let-7d promoter in HCC [15]. Here, we charaterize the promoter and transcription start site(s) of MC-let-7a-1~let-7 in GBM U87 and U251 cells by 5′ Rapid Amplification of cDNA Ends (5′RACE) assays using total RNA as templates, gene-specific primers GSP1-1 for the synthesis of first strand cDNA, and GSP2-1 and GSP2-2 for nested PCR (Figure 2A). A ~700 bp product was amplified (Figure 2B) and cloned. Sequence analysis of twenty 5′RACE products (10 products for each cells) revealed that the TSSs of MC-let-7a-1~let-7d in U87 and U251 are located about 28 bp and 40 bp, respectively, downstream of the TSSs found in HepG2 cells (Figure 2C). These results suggested that MC-let-7a-1~let-7d uses similar promoter regions in HCC and GBM with different TSSs.

In addition, we compared the promoter activity of the highly conserved region around the TSSs. Our results showed that the promoter regions of MC-let-7a-1~let-7d exhibited similar property in GBM (U87 cells) as compared to that of the liver L02 cells. As shown in Figure 2D, truncation analysis revealed that promoter (−540bp to +1bp) exhibited maximal activity. In addition, the presence of a suppression element from +1317 to +1 and an activator from −540 to −227 were detected in both U87 cells and L02 cells.

### MYC failed to inhibit the expression of human MC-let-7a-1~let-7d microRNA cluster in GBM cells

**Figure 3:**
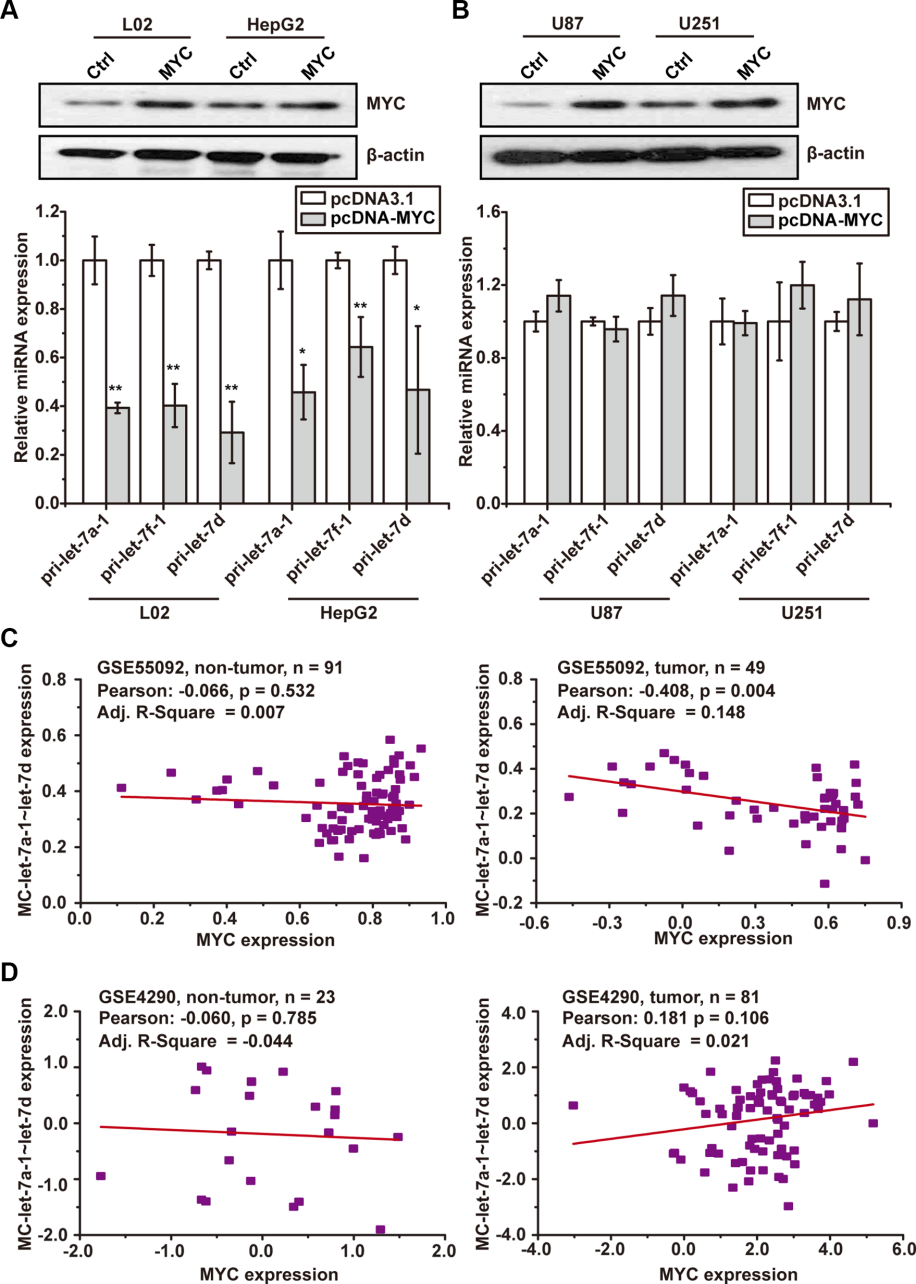
MYConcoproteindown-regulates MC-let-7a-1~let-7d in HCC, but not in GBM (**A**) forced expression of MYC significantly down-regulated the MC-let-7a-1~let-7d expression in L02 and HepG2 cells. (**B**) forced expression of c-Myc failed to down-regulate the MC-let-7a-1~let-7d expression in U87 and U251 cells. (**C**) the expression correlativity of MYC and MC-let-7a-1~let-7d in HCC patient samples (right panel) and non-tumor control (left panel) according to GSE55092 data set. (**D**) the expression correlativity of MYC and MC-let-7a-1~let-7d in GBM patient samples (right panel) and non-tumor control (left panel) according to GSE4290 data set. Pearson correlation is calculated to measure the strength of a linear association between the expressions of two genes. R-square is the coefficient of linear regression which measures the linearity between the expressions of two genes. *p < 0.05; **p < 0.01.

To investigate whether MYC differentially regulates the transcription of MC-let-7a-1~let-7d in HCC and GBM, we over-expressed MYC in HepG2, L02, U87, and U251 cells. As expected, forced expression of MYC significantly down-regulated MC-let-7a-1~let-7d expression only in L02 and HepG2 cells (Figure3A), but not in U87 and U251 cells (Figure 3B).

We further validated these findings in HCC- and GBM-patient samples. Pearson's correlation and Linear Regression analysis were employed using data from GSE55092 (HCC, tumor = 49, non-tumor = 91) and GSE4290 (GBM, tumor = 81, non-tumor = 23). The converse relationship of MC-let-7a-1~let-7d and MYC was only observed in HCC samples (Figure 3C, right panel, Pearson = 0.408, *p* = 0.004), but not in normal liver samples (Figure 3C, left panel, Pearson = −0.066, *p* = 0.532), normal brain samples (Figure 3D, left panel, Pearson = −060,*p* = 0.785) or glioblastoma samples (Figure 3D, right panel, Pearson = 0.181, *p* = 0.106).

### MYC failed to down-regulate the promoter activity of MC-let-7a-1~let-7d cluster in GBM

**Figure 4:**
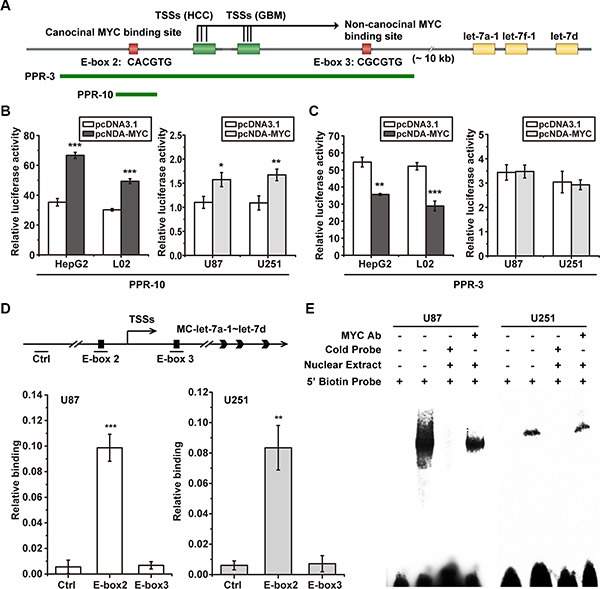
MYC failed to bind to the repressive non-canonical E-box 3 in GBM cells (**A**) schematic representation of the location of MC-let-7a-1~let-7d promoter PPR-3 and PPR-10 and the E-boxes. (**B**) overexpression of MYC increased PPR-10 activity in both HCC cells HepG2 and L02 and GBM cells U251 and U87. (**C**) overexpression of MYC decreased PPR-3 activity in HCC cells HepG2 and L02, whereas failed to decrease its activity in GBM cells U251 and U87. (**D**) ChIP assays were performed to quantify the MYC binding quantum on E-box 2 and E-box 3. Schematic representation of the location of Ctrl, E-box 2, and E-box 3 (upper panel) is shown. The Real-time PCR results were summarized as relative binding (MYC ChIP relative to input ChIP). The E-box 2 region was significantly enriched, whereas the E-box 3 region was not enriched both in U87 and U251. (**E**) EMSA and supershift assay analysis of MYC binding to E-box 3 in U87 and U251. Competition assays were performed adding to 100-fold molar unlabeled E-boxes. Shift bands were observed by adding nuclear extract form both U87 and U251, whereas supershift bands was not observed by adding nuclear extract form U87 and U251 with MYC antibody.

Our previous study showed that MYC down-regulates the transcription of MC-let-7a-1~let-7d in HCC, and identified two functional MYC binding E-box elements (E-box 2, 3) [15]. We showed that over-expression of MYC significantly reduced the expression of MC-let-7a-1~let- 7d primary transcript through the non-canonical E-box 3, in L02 and HepG2 cells. To examine the transcriptional regulation in GBM, we first determined the effect of MYC in MC-let-7a-1~let-7d promoter PPR-10 which only contains canonical E-box 2 (Figure 4A). Our results indicated that over-expression of MYC activated luciferase activity of PPR-10 in all the four cells lines HepG2, L02, U87, and U251, suggesting the similar function of basal activity of canonical E-box 2 (Figure 4B). Furthermore, over-expression of MYC significantly repressed the activity of PPR-3 which contains both canonical E-box 2 and non-canonical E-box 3 in HepG2 and L02 cells, however, it could not change the activity of PPR-3 in GBM U87 and U251 cells (Figure 4C). These results suggested a defective function of the non-canonical E-box 3 in GBM.

### Defective MYC and E-box 3 binding in GBM

We determined the binding ability of MYC to E-box 3 in GBM U87 and U251 cells by ChIP assays to quantify the MYC binding quantum on E-box 2 and E-box 3. The results from Real-time PCR experiments were summarized and shown as relative binding (MYC ChIP relative to input ChIP). As shown in Figure 4D, strong MYC binding was observed in E-box 2 but not in E-box 3, suggesting the absence of MYC and E-box 3 binding in GBM.

Furthermore, EMSA assays and super-shift analysis were performed to confirm the loss of MYC binding to E-box 3 in U87 and U251 cells. Competition assays were conducted by adding 100-fold molar unlabeled E-boxes. As shown in Figure 4E, E-box 3 showed shift bands by adding nuclear extract from U87 and U251 cells. However, supershift bands were not observed by adding nuclear extract form U87 and U251 with MYC antibody. These results confirmed that MYC onco-protein could not bind to E-box 3 in GBM.

## DISCUSSION

MYC is an evolutionarily conserved E-box binding transcription factor. It is pathologically activated in many human malignancies [41]. The expression of MYC is important in cell signaling pathways such as cell growth/proliferation, metabolism and apoptosis [42]. It has been reported that MYC expression is elevated in almost all cancers including both HCC and GBM [18, 41]. As compared to a nearly undetectable level in normal brain tissues, MYC is expressed in 80.6% of primary glioblastoma samples [43, 44], and in 73% of astrocytomas [45]. The wide spread repression of miRNAs including let-7 by MYC and its contribution to tumorigenesis has also been clearly demonstrated [46].

**Figure 5:**
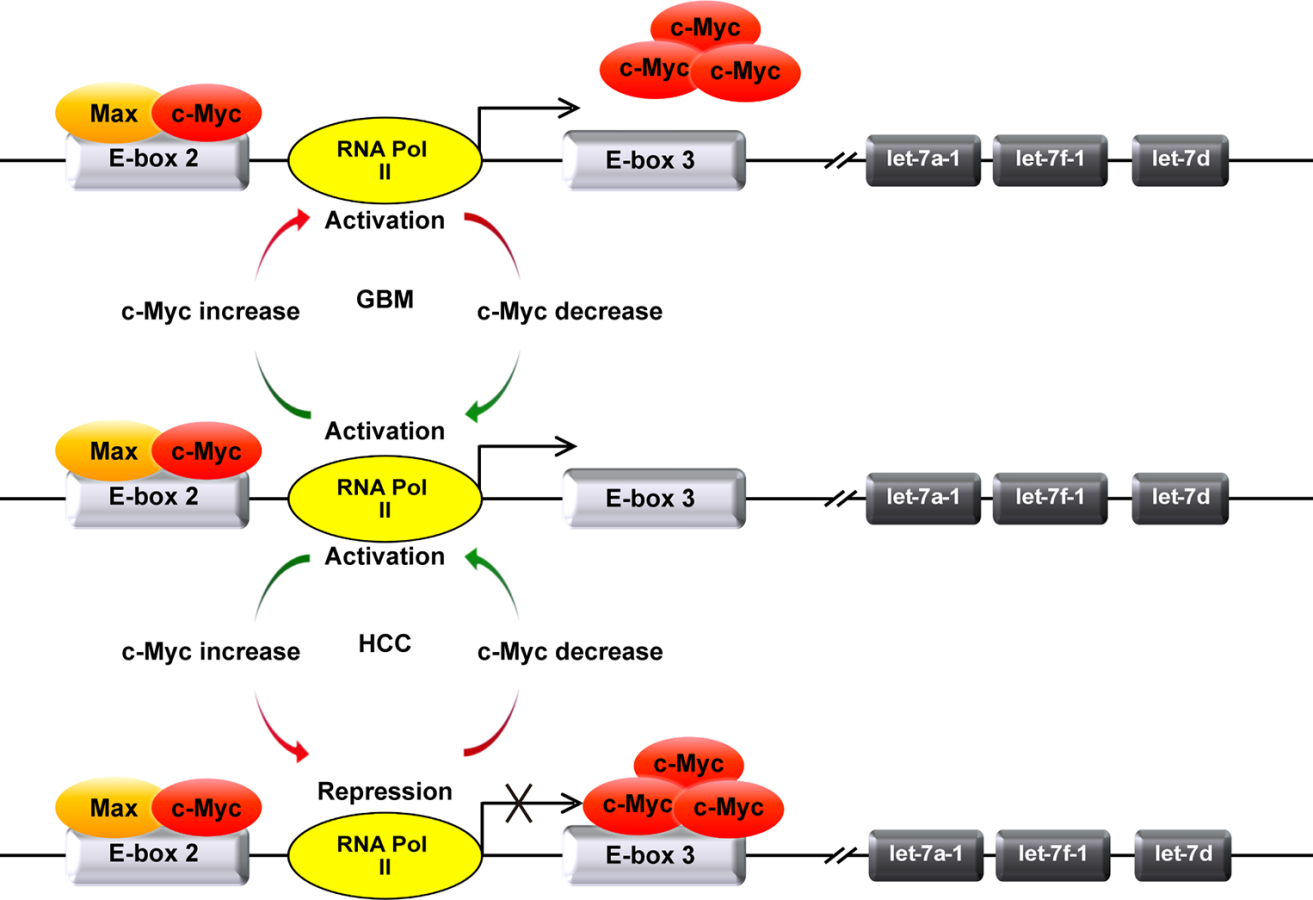
Model depicts the mechanism of the differential transcriptional regulation of MC-let-7a-1~let-7d in GBM and HCC In HCC, a small amount of c-Myc preferentially dimerizes with Max and binds E-box 2 to maintain the basal activity of MC-let-7a-1~let-7d promoter. The extra c-Myc will bind to E-box 3 and repress the promoter activity of MC-let-7a-1~let-7d. In GBM, likewise, a small amount of c-Myc preferentially dimerizes with Max and binds E-box 2 to enhance the transcription of MC-let-7a-1~let-7d. Differently, the extra c-Myc failed to bind to non-canonical E-box 3 to inhibit the expression of MC-let-7a-1~let-7d in GBM cells.

Previously, we reported that MYC inhibited the transcription of MC-let-7a-1~let-7d in HCC [15]. We proposed that MYC exhibited dual functions in the transcriptional regulation of MC-let-7a-1~let-7d. As shown in Figure 5, in non-cancerous L02 cells with a low level of MYC, MYC dimerizes with Max and bind to the canonical E-box 2 to activate and maintain the basal expression level of let-7. However, in cancerous HepG2 cells with elevated MYC, the extra MYC binds to the non-canonical E-box 3 to suppress the transcription of MC-let-7a-1~let-7d. These mechanisms enable cancer cells to maintain a high level of MYC and a low level of let-7 miRNAs simultaneously in HCC.

In present study, we reported that MC-let-7a-1~let-7d is not down-regulated in GBM due to the loss of MYC and E-box 3 binding. The detailed molecular mechanism for this defect is not known at present. One explaination is that the binding of MYC to E-box 3 requires additional unknown intermediary trans-acting factors that are defective or absent in GBM.

In the promoter truncation assay, we found that the −540 bp to +1bp region of MC-let-7a-1~let-7d promoter exhibited maximal promoter activity, while longer promoters PPR-1~4 showed decreased promoter activity. These results indicated the presence of suppression elements downstream of the transcription initiation site. Indeed, our study showed that MYC also acts as a transcription suppressor by binding to the non-canonical binding site E-box 3 located downstream of TSSs. However, it is not clear whether and how MYC bind to the non-canonical DNA sequence to suppress the promoter activity. Results from our study inferred that MYC might not bind to E-box3 directly, but is recruited to the promoter by yet to be identified factor, which is present in HCC, but either defective or absent in GBM. Identification and characterization of this MYC-E- box3 binding partner will have important implications as therapeutic target and biomarkers for GBM and cancers.

It has been reported that the expression of let-7 family members are increased during neural differentiation and let-7 members play important roles in neural stem cell proliferation and differentiation [47–50]. Lee *et al* showed that over-expression of let-7 miRNAs could reduce the proliferation and migration of GBM cells, as well as the tumor size in nude mice xenograft transplanted GBM [51]. Forced expression of let-7 miRNA reduced expression of pan-RAS, N-RAS, and K-RAS [51], suggesting that let-7 played important tumor suppressor roles in GBM. Our *in silico* and wet-lab findings suggest that let-7 escapes MYC inhibition in GBM. It is not known whether the co-existence of high levels of onco-MYC and tumor suppressor-let-7 had any impact in the carcinogenesis of brain tumors. As let-7 miRNAs could inhibit the expression of MYC, and how MYC escapes inhibition by let-7 in GBM is also not understood. The existence of recently discovered competing endogenous RNA (ceRNA), and whether they play a role requires further investigations [52, 53].

The present study provided a model to explain the the differential transcriptional regulation of tumor suppressor let-7 by oncogene MYC in glioblastoma and hepatocellular carcinoma. These results suggest a potential molecular mechanism for understanding the complexity of molecular regulatory network. The potential implications in cancer treatment warrant further investigations.

## MATERIALS AND METHODS

### Cell lines and cell culture

Human HCC cell line HepG2 and immortalized human liver cell line L02 were obtained from American Type Culture Collection (ATCC, Manassas, VA). Human high-grade glioma cell lines (U-87 MG, U-251 MG, and U-373 MG), human primitive neuroectodermal PFSK-1 cells, and human low-grade glioma SW-1088 cells were also obtained from ATCC. All these cell lines were cultured in Dulbecco's modified eagles medium (DMEM, Invitrogen) supplemented with 10% fetal bovine serum (FBS, Gibco) at 37°C in a humidified atmosphere with 5% CO_2_ as our previous report [15, 54].

### Tissue samples

Three adjacent normal human brain tissue (NBT) specimens were collected from 3 male GBM patients with 23-, 34- and 35-year-old respectively at Sun Yat-sen University Cancer Center. Patients' consent and approval from Sun Yatsen University Cancer Center Institute Research Ethics Committee were obtained for the use of these clinical materials. For each sample, total RNA were purified by using AllPrep Mini Kit (Qiagen, Cat number 80004) according to the manufactures' instructions.

### Genomic DNA extraction, RNA extraction, cDNA synthesis and Real-Time PCR

For genomic DNA extraction, total cell genomic DNA was extracted using the DNeasy Blood & Tissue (Qiagen), following the manufacturer's instructions. For RNA extraction, total RNA was extracted using Trizol (Invitrogen), according to the manufacturer's instructions. For cDNA synthesis, 2 μg total RNA was reverse transcribed by the SuperScript™ III First-Strand Synthesis System (Invitrogen) after DNase I treatment (Invitrogen). Quantitive Real-Time polymerase chain reaction (qRT-PCR) was subsequently performed in triplicate with a 1:10 dilution of the resultant cDNA using the Brilliant Brilliant^®^ II SYBR^®^ Green QPCR Master Mix (Stratagene) on the Applied Biosystems 7500 Real-Time PCR System Instrument (Applied Biosystems). All mRNA quantification data were normalized to GAPDH. Ct-value for each sample was calculated with the ΔΔCt-method and results were expressed as 2^−ΔΔCT^ [55].

### Protein Extraction and Western blot analysis

Cells were seeded onto a 6-well plate at a density of 10^6^ cells/well. Cells were lysed for 30 min on ice in RIPA lysis buffer (Bioteke) with protease inhibitor cocktail. Protein concentration was determined by Bio-Rad protein assay kit (Bio-Rad). Fifty ug proteins were separated by 12% SDS polyacrylamide gels (SDS-PAGE) and transferred onto PVDF membranes. Membranes were probed with specific antibodies includingMYC (1:2000; Abcam) or β-actin (1:3000; Abcam) respectively. After washing, membranes were incubated with HRP-conjugated secondary antibody (1:5000; Abcam). Membranes were exposed using the enhanced chemiluminescence (ECL) substrate kit (Thermo) and imaged using the ImageQuant™ RT ECL™ system (GE Healthcare). The expression level of β-actin was also determined and served as an internal control.

### 5′ Rapid Amplification of cDNA Ends (5′ RACE)

The 5′ RACE System for Rapid Amplification of cDNA Ends, Version 2.0 (Invitrogen) was used according to the manufacturer's instructions. Briefly, cDNA was synthesized using the MC-let-7a-1~let-7d Specific Primers GSP1-1 (Supplementary Table S2). Primary amplification was carried out with abridged anchor primer (AAP, provided in the kit) and GSP2-1 primer (Supplementary Table S2). Nested PCR was performed with abridged universal amplification primer (AUAP, provided in the kit) and GSP2-2 (Supplementary Table S2) using TaKaRa LA Taq^®^ with GC Buffer I (TaKaRa). The 5′RACE PCR products were resolved on 1% agarose gel and stained by EtBr. The bands were excised, cloned into pGL3 Basic Vector (Promega) via restriction endonuclease Mlu I sites and sequenced.

### Constructions of promoter reporter, and MYC expression plasmids

A 1.9 kb MC-let-7a-1~let-7d promoter was amplified by PCR using PrimeSTAR™ HS DNA Polymerase (TaKaRa) and cloned into pGL3 Basic Vector (Promega) between Xho I and Hind III sites using primers PPR-1 as listed in Supplementary Table S2. This plasmid was named as PPR-1. Subsequent promoter truncations were generated from this template and cloned in a similar manner using the primers in Supplementary Table S2. To construct MYC expression vector, human MYC open reading frame (ORF) was amplified using MYC primers as indicated in Supplementary Table S2 and inserted into the pcDNA3.1(+) vector (Invitrogen) between Hind III and BamH I sites. The sequence and orientation of all of the inserts were confirmed by sequencing.

### Luciferase reporter assays

HepG2 cells, L02 cells, U87 cells or U251 cells cells were plated at a density of 50,000 cells per well in a 24-well plate at 24 hrs before transfection. Cells were co-transfected with 250 ng MC-let-7a-1~let-7d promoter/firefly luciferase reporter plasmids and 5 ng pRL-TK Renilla plasmids (Promega) using FuGENE^®^ HD Transfection Reagent (Promega). For gain and loss-of-function experiments, 750 ng MYC expression plasmids (c-Myc) or its control vectors (pcDNA3.1) were transfected. After 48 h post-transfection, cells were either lysed in Trizol (Invitrogen) for RNA extraction or in passive lysis buffer (Promega) for luciferase assay measured with the Dual-Luciferase Reporter Assay System (Promega) using the TD-20/20 Luminometer (Turner Designs). The relative luciferase activities were determined by calculating the ratio of firefly luciferase activities over *Renilla* luciferase activities.

### Chromatin immunoprecipitation (ChIP)

ChIP assays were carried out by using an EZ-ChIP assay kit (Millipore), in accordance with the manufacturer's instructions. In brief, U87 and U251 cells were grown to 90% confluence and added 1% formaldehyde at room temperature for 10 min. The cross-link reaction was quenched with 0.125 M glycine for 5 min at room temperature. Cells were then washed, scraped, and resuspended in 1 ml of lysis buffer. DNA was sonicated into around 400-bp pieces at 4°C by using Sonics UibraCellsTM (XINCHEN). Supernatants were recovered by centrifugation and precleared for 1 h at 4°C with 60 μl of protein G-agarose. Then 10 μl (1%) of supernatant was removed as input. Immunoprecipitations were performed overnight with MYC antibody (Abcam, 9E11, ChIP Grade, 6 ug) or IgG antibody (provided in kit, 1 ug). The immune complexes were captured by incubation with 60 ulof protein G-agarose for 2 h at 4°C. The immunoprecipitates were washed sequentially with wash buffers. After that, the immunoprecipitates were eluted from the protein G-agarose by incubating with elution buffer (1% SDS, 100 mMNaHCO3). DNA-protein complexes were reversely cross-linked by a high salt solution at 65°C for 5 h. RNA and protein were eliminated by treating with 10 ug of RNase A at 37°C for 30 min and then with protease K for 2 h at 45°C. Finally, DNA was purified by using the spin column provided in the ChIP kit and eluted with 50 ul of elution buffer. qRT-PCR was performed by using Brilliant Brilliant^®^ II SYBR^®^ Green QPCR Master Mix (Stratagene) on the Applied Biosystems 7500 Real-Time PCR System Instrument (Applied Biosystems). The primer pairs used for PCR analysis are shown in Supplementary Table S2. All data were normalized to input as the previous report [56].

### Electrophoretic mobility shift assay (EMSA)

Nuclear extracts from U87 and U251 cells were prepared in accordance with the manufacturer's protocol (NE-PER® Nuclear and Cytoplasmic Extraction Reagents, Pierce). Complementary oligonucleotide pairs corresponding to the E-box 2 and E-box 3 embedded in the promoter region of *MC-let-7a-1~let-7d* and 5′ end-labeled with biotin by Invitrogen. The oligonucleotide sequences were shown in Supplementary Table S2. EMSA assay was performed by using a LightShift^®^ Chemiluminescent EMSA Kit (Pierce). Binding reaction with 10 μg of HepG2 nuclear extracts and 100 fmol 5′ Biotin-labeled oligonucleotide was carried out in accordance with the manufacturer's instructions. For the competition assay, a 100-fold molar excess of unlabeled oligonucleotide was added to the binding reaction mixture as a specific competitor. For antibody-supershift assay, a nuclear extract was pre-incubated with a 3 μl MYC antibody (Abcam) before adding it to the binding reaction. DNA/protein complexes were separated on a pre-electrophoresed 6% polyacrylamide gel in 0.5 × TBE, transferred to a nylon membrane, and cross-linked at 120 mJ/cm^2^ for 1 minute and detected by chemiluminescence, in accordance with the manufacturer's directions. Membranes were exposed by using the enhanced chemiluminescence (ECL) substrate kit (Thermo) and then were photo-documented.

### Statistical analysis

Data were shown as means ± SEM. Statistical analyses for detection of significant differences between the control and experimental groups were carried out using unpaired two-tailed Student's *t* test (*p ≤ 0.05; **p ≤ 0.01; ***p ≤ 0.001).

## SUPPLEMENTARY MATERIALS TABLES



## Abbreviations

HCC: Hepatocarcinoma
GBM: glioblastoma
ChIP: chromatin immunoprecipitation
miRNAs: microRNA
NBT: normal brain tissues
NLT: normal liver tissues
5′RACE: 5′ Rapid Amplification of cDNA Ends
GSP: gene-specific primers
ATCC: American Type Culture Collection
DMEM: Dulbecco's modified eagles medium
FBS: fetal bovine serum
EMSA: Electrophoretic Mobility Shift Assay
ECL: chemiluminescence
SDS-PAGE: sodium dodecyl sulfate-polyacrylamide gel electrophoresis
PVDF: polyvinylidene difluoride
