# Supplementary material for: Loss of MYC and E-box3 binding contributes to defective MYC-mediated transcriptional suppression of human MC-let-7a-1~let-7d in glioblastoma
Source: Oncotarget. 2016 Jul 9;7(35):56266–78. doi: 10.18632/oncotarget.10517 (PMC5302913; doi:10.18632/oncotarget.10517)
Supplement: Supplementary file 1 [file oncotarget-07-56266-s001.pdf]

Loss of MYC and E-box3 binding contributes to defective MYC-mediated transcriptional suppression of human MC-let-7a-1~let-7d in glioblastoma

Supplementary Materials

Supplementary Table S1: let-7 family expression in glioblastoma detected by 11 different groups

mature miRNA	Zhou et al, 2010	Slaby et al, 2010	Erns et al, 2010	Wu et al, 2009	Huse et al, 2009	Sasayama et al, 2009	Malzkorn et al, 2009	Conti et al, 2009	Silber et al, 2008	Godlewski et al, 2008	Ciafre et al, 2005	SUM
hsa-let-7a	0	0	0	0	0	0	0	0	0	0	0	0
hsa-let-7a*	0	0	0	0	0	0	0	0	0	0	0	0
hsa-let-7a-2*	0	0	0	0	0	0	0	0	0	0	0	0
hsa-let-7b	0	0	0	0	0	0	0	0	0	0	0	0
hsa-let-7b*	0	0	0	0	0	0	0	0	0	0	0	0
hsa-let-7c	0	0	0	0	0	0	0	0	0	0	0	0
hsa-let-7c*	0	0	0	0	0	0	0	0	0	0	0	0
hsa-let-7d	0	0	0	−1	0	0	0	0	0	0	0	-1
hsa-let-7d*	0	0	0	0	0	0	0	0	0	0	0	0
hsa-let-7e	0	0	0	0	0	0	0	0	0	0	0	0
hsa-let-7e*	0	0	0	0	0	0	0	0	0	0	0	0
hsa-let-7f	0	0	0	−1	0	0	0	0	0	0	0	−1
hsa-let-7f-1*	0	0	0	0	0	0	0	0	0	0	0	0
hsa-let-7f-2*	0	0	0	0	0	0	0	0	0	0	0	0
hsa-let-7g	0	0	0	−1	0	0	0	0	0	0	0	−1
hsa-let-7g*	0	0	0	0	0	0	0	0	0	0	0	0
hsa-let-7i	0	0	0	0	0	0	0	0	0	0	0	0
hsa-let-7i*	0	0	0	0	0	0	0	0	0	0	0	0
hsa-miR-98	0	0	0	0	0	0	0	0	0	0	0	0
hsa-miR-202	0	0	0	0	0	0	0	0	0	0	0	0
hsa-miR-202*	0	0	0	0	0	0	0	0	0	0	0	0

Annotation: "0" indicates no significant change; "-1" indicates downregulation.

SupplementaryTable S2: Primers used in this study

name	Sense primer (5′ to 3′)	Anti-sense primer (5′ to 3′)	Application
GAPDH	GAATCTACTGGCGTCTTCACC	GTCATGAGCCCTTCCACGATGC	Real-Time PCR
pri-let-7a-1	ACAGGAAACCAGGATTACCGAG	ACAACCTACTACCTCATCCCACAG	Real-Time PCR
pri-let-7f-1	GCATCTGCCAAGTAGAAGACCAG	CCAAAGCAAAGTAGCAAGGAAAC	Real-Time PCR
pri-let-7d	TACTTTCCATTCCAGAAGAAA ACATT	ATAATGCAGCAAGTCTACTCCTCAG	Real-Time PCR
c-Myc	GGCTCCTGGCAAAAGGTCA	CTGCGTAGTTGTGCTGATGT	Real-Time PCR
PPR-1	GACACTCGAGCAAACTGAGGC ACAGAACTCC	ATATAAGCTTAATAAAA CCAGACCCCGCCAAAC	Promoter Cloning
PPR-2	GACACTCGAGCAAACTGAGGC ACAGAACTCC	TCCCAAGCTTTTTCCTGTCTGCC CGCCCTCTCG	Promoter Cloning
PPR-3	GACACTCGAGCAAACTGAGGC ACAGAACTCC	TCCGAAGCTTCCGAGTC GCAAATCTCCAAGC	Promoter Cloning
PPR-4	GACACTCGAGCAAACTGAGGC ACAGAACTCC	GGAAAAGCTTGGTAAGGAATG TGAAGAATGACCC	Promoter Cloning
PPR-5	GACACTCGAGCAAACTGAGGC ACAGAACTCC	GCACAAGCTTAGGACCCGGAT GGAGGC	Promoter Cloning
PPR-6	AGCCCTCGAGTAG-GAAAGAAGGAGATGGTATTG	GCACAAGCTTAGGACCCGGAT GGAGGC	Promoter Cloning
PPR-7	AATCCTCGAGCTTCAC-CAATGGGCTTGC	GCACAAGCTTAGGACCCGGAT GGAGGC	Promoter Cloning
PPR-8	GGAGCTCGAGGGAG-CAAAGCAGCCG	GTGGAAGCTTCCAGGCCC TGCCCTTT	Promoter Cloning
PPR-9	GACACTCGAGCAAACTGAGGC ACAGAACTCC	GGTCAAGCTTGCCCACCAATA CCATCTC	Promoter Cloning
PPR-10	GGAGCTCGAGGGAG-CAAAGCAGCCG	GTGGAAGCTTCCAGGCCC TGCCCTTT	Promoter Cloning
c-Myc ORF	AGCTAAGCTTACCAT GGATTTTTTTCGGGTAGTGG	TTACGGATCCTTACGC ACAAGAGTTCCGTAGC	c-Myc Cloning
EMSA E-box 3	GGGAGGGGTGCCGCGTGGCCG CCGCGGGG	CCCCGCGGCGGCCACGC GGCACCCCTCCC	EMSA
EMSA Bio-E-box 3	Biotin-GGGAGGGGTGCCGCGT GGCCGCCGCGGGG	Biotin-CCCCGCGGCGGCCACGC GGCACCCCTCCC	EMSA
GSP1-1	GTTATCAATGTCAGCACC		5′RACE
GSP2-1	TTCCACGCGTACCGAGTC GCAAATCTCCAAGC		5′RACE
GSP2-2	CCCCACGCGTC GCACTTCCTACCTTCCCTCCTT		5′RACE
